# Sex differences in thermoregulation in mammals: Implications for energy homeostasis

**DOI:** 10.3389/fendo.2023.1093376

**Published:** 2023-03-08

**Authors:** Carlos Fernández-Peña, Alfonso Reimúndez, Félix Viana, Victor M. Arce, Rosa Señarís

**Affiliations:** ^1^ Institute of Neuroscience, University Miguel Hernández (UMH)-CSIC, Alicante, Spain; ^2^ Department of Physiology, CIMUS, University of Santiago de Compostela, Santiago de Compostela, Spain

**Keywords:** sex, thermoregulation, energy homeostasis, estrogens, progesterone, BAT, browning

## Abstract

Thermal homeostasis is a fundamental process in mammals, which allows the maintenance of a constant internal body temperature to ensure an efficient function of cells despite changes in ambient temperature. Increasing evidence has revealed the great impact of thermoregulation on energy homeostasis. Homeothermy requires a fine regulation of food intake, heat production, conservation and dissipation and energy expenditure. A great interest on this field of research has re-emerged following the discovery of thermogenic brown adipose tissue and browning of white fat in adult humans, with a potential clinical relevance on obesity and metabolic comorbidities. However, most of our knowledge comes from male animal models or men, which introduces unwanted biases on the findings. In this review, we discuss how differences in sex-dependent characteristics (anthropometry, body composition, hormonal regulation, and other sexual factors) influence numerous aspects of thermal regulation, which impact on energy homeostasis. Individuals of both sexes should be used in the experimental paradigms, considering the ovarian cycles and sexual hormonal regulation as influential factors in these studies. Only by collecting data in both sexes on molecular, functional, and clinical aspects, we will be able to establish in a rigorous way the real impact of thermoregulation on energy homeostasis, opening new avenues in the understanding and treatment of obesity and metabolic associated diseases.

## 1 Introduction: Importance of body temperature homeostasis

Temperature is a critical factor for the development and survival of all organisms. Thermal homeostasis requires the precise regulation of numerous physiological and behavioural mechanisms. Animal species have developed different strategies to maintain an internal temperature which may allow an efficient function of cells and organs, despite a changing external environment (1). Depending on their processes of thermoregulation, organisms are divided into ectotherms and endotherms (2). In ectotherms, internal sources of heat are negligible, and body temperature relies on environmental heat. They are usually poikilotherms, although some species can maintain a homeothermic state by using behavioural mechanisms. In contrast, endotherms use internal metabolic processes to generate heat and usually maintain a narrow range of core body temperature (Tc), homeothermy.

Endothermy, the internal production of heat, has been very important during evolution. It allowed species, like birds and mammals, to live rather independently from external sources of heat, thus enlarging their ecological niches. However, this mode of living requires the consumption of large amounts of food, providing sufficient energy to sustain a high and stable Tc. Fluctuations of environmental temperatures turn on thermoregulatory mechanisms to defend homeothermy, i.e. heat-conserving and heat producing responses, when exposed to low temperatures and heat-dissipation mechanisms when temperature increases. Extreme external temperatures that overwhelm the normal thermoregulatory mechanisms or, alternatively, disruptions in the normal functioning of the thermoregulatory system will produce hyperthermia or hypothermia, which may be life-threatening.

Increasing evidence has highlighted the intimate interlink between thermal and energy homeostasis. The maintenance of homeothermy requires a fine regulation of food intake, heat production, heat conservation or dissipation and energy expenditure, which will heavily affect energy metabolism and energy balance. Although basic mechanisms responsible for maintaining the Tc in mammals are similar in males and females, differences in anatomic, hormonal, and other sex-dependent characteristics greatly influence several aspects of thermoperception, thresholds of activation of thermoregulatory effectors and body temperature levels and set points. The present review focuses on sexual differences in thermoregulation and their implications for energy homeostasis. To do so, we will first provide a brief overview of the variables governing temperature regulation, which will serve to provide a background to evaluate potential sex differences. We finally discuss how sex-dependent characteristics in thermal responses may induce metabolic differences between males and females, with a clear physiological, clinical, and therapeutic relevance.

## 2 Sex differences in core body temperature and thermoneutral zone

Tc in endothermic homeotherms is the result of an increased generation of basal heat, also called obligatory or basal thermogenesis, which is mainly dependent on thyroid hormones, and the development of regulatory processes to maintain this internal temperature around a set point(s), through thermogenic and heat dissipative processes. Temperature is detected at the skin and in the core by thermal sensors, which send afferent information to the central thermoregulatory centre in the preoptic area of the hypothalamus (POA) and to sensory and other cortical areas to orchestrate the activation of thermoregulatory mechanisms. Warm and cold sensitive neurons are also found in the POA (3, 4).

In most mammals, including humans, average Tc is around 37 °C and oscillates between day and night following a circadian rhythm with a period close to 24 hours. The circadian system modulates metabolic heat production and heat loss to generate the Tc rhythm (5). In nocturnal rodents (i.e., the great majority), the uppermost Tc levels are reached during the night (active phase) and the lowermost values are attained during the daytime (inactive phase). The opposite occurs in humans, due to our diurnal condition (6). In both, rodents and humans, there is a diurnal variation of ≈ 1 °C (5, 6). Tc circadian oscillations are mainly regulated by neurons in the suprachiasmatic nucleus of the hypothalamus (SCN), the master clock of the organism, which have been shown to project to the thermoregulatory circuits in the POA to imprint the circadian fluctuations (7, 8).

Finally, sex-specific differences are observed in Tc levels in laboratory animals (9, 10) and in humans (6). Furthermore, Tc experience cyclic changes throughout the ovarian cycle in mammalian females, suggesting that sexual hormones regulate thermal homeostasis.

**Figure 1:**
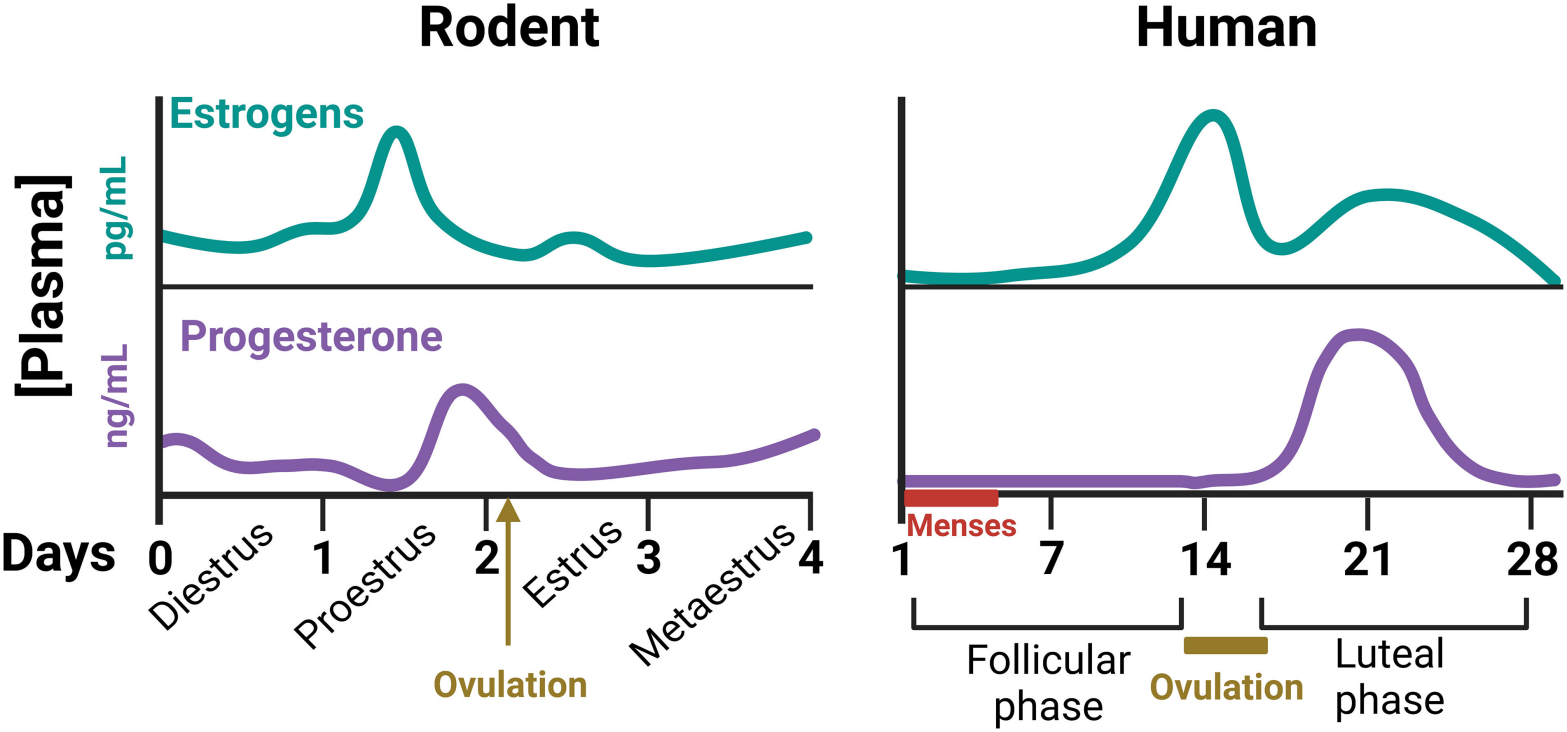
Schematic representation of plasma concentrations of gonadal steroids throughout the ovarian cycle of female rodents and humans (11, 12). These cyclic changes of ovarian hormones not only control the development of the ovarian follicles, but many other body functions, including thermal homeostasis.

The ovarian cycle is essential for periodic production of mature ova through ovulation but encompasses many other cyclic events in reproductive tissues (uterus, vagina, external genitalia,…). Maintenance of the ovarian cycle mainly depends on the coordinated function of the hypothalamic-pituitary-ovarian axis. Briefly, secretion of gonadotropin-releasing hormone (GnRH) from the hypothalamus induces the synthesis and liberation of the gonadotropins, luteinizing hormone (LH) and follicle stimulating hormone (FSH) by pituitary gonadotrophs. Gonadotropins act on cells of the ovarian follicles, regulating the secretion of estrogens and progesterone. Finally, estrogens and progesterone drive the development of ovarian follicles and exert a large variety of reproductive and non-reproductive actions in the female organism (11). Significant differences in ovarian cyclicity exist between rodents and humans. In rodents, the ovarian cycle is linked to changes in reproductive behavior and lasts 4-5 days (commonly called estrous cycle). It is generally divided into four stages: proestrus, estrus, metaestrus and diestrus, with cyclic changes of sexual hormones (**Figure 1**). Estrus is the period of the cycle during which ovulation occurs and mating behavior is displayed (12). In women, ovarian cycle lasts approximately 28 days, and is divided into a follicular phase (with high levels of estrogens), and a luteal phase (with high levels of progesterone). Cyclic ovarian hormonal changes are also responsible in women for the presence of the menstrual cycles, i.e., the regular appearance of menses due to the shedding of the endometrium (11) (**Figure 1**).

**Figure 2:**
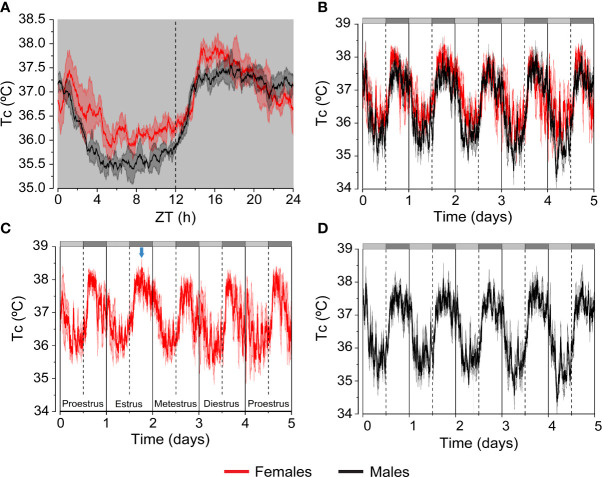
Differences in Tc between female and male mice. Continuous telemetric recordings of Tc in C57B6/J female and male mice housed at 21 °C. Mice were exposed to constant darkness (D/D) for 5 days to analyze circadian fluctuations. **(A)** The graph represents daily average of the Tc values recorded during the last 3 days. **(B–D)** The graphs represent daily variation of the Tc values recorded during 5 days in females and males. Subjective day and subjective night are indicated by the light grey and dark grey bars, respectively. The blue arrow points at the characteristic shape of Tc during estrus. n= 4-5 mice/group of 5-8 months of age. Results are represented as mean ± SEM. These graphs were elaborated from data of experiments reported in Reimúndez et al., 2022 (14).

In rodents and humans Tc levels are higher in the feminine sex (0.2-0.5 °C higher) (6, 9, 10, 13) (**Figures 2A, B**). Furthermore, Tc recordings during several days in females reflect the changes of this variable across the ovarian cycle. Thus, Tc in female mice shows sustained high levels during the dark phase of estrus versus the typical biphasic profile of the other phases of the estrous cycle. In contrast, Tc in male mice display a consistent and constant pattern (10) (**Figures 2C, D**). In women, there is a notable regulation of Tc throughout the ovarian cycle with an increase of 0.3-0.7 °C in the luteal phase, in comparison with the lower and constant levels in men (6). The physiological significance of the higher levels of Tc in females is not clear, but it may serve to create an accurate environment for implantation and survival of the embryo (6).

Although the mechanism(s) underlying the differences in Tc between sexes are still not well established, gonadal hormones are considered as main factors. Among them, progesterone seems to play the most crucial role as thermogenic signal, although, the progesterone:estrogen ratio is also important for modulating Tc in females (6, 10). Sex hormones are proposed to influence Tc by direct action on hypothalamic POA neurons. In fact, receptors for progesterone, estrogens and testosterone are expressed in this hypothalamic area and regulatory effects of these hormones have been described, especially, on temperature-sensitive neurons (15–17). However, the major effect at the central level is exerted by progesterone. This hormone shifts the thermoregulatory set point(s) in the hypothalamus towards higher temperatures. This results in a higher Tc, as it can be observed in the luteal phase of the human ovarian cycle, with very high levels of circulating progesterone (6, 18) (**Figure 1**). In rodents the effect is not so evident because they do not have a real luteal phase, but the increase of plasma progesterone concentrations during the end of proestrus and beginning of estrus may be involved in the elevated Tc levels of estrus (19) (**Figure 1**). Nevertheless, the cellular and molecular mechanisms and circuitry to induce progesterone’s thermogenic effects are still poorly understood. Estrogens and testosterone are proposed to participate principally in the peripheral regulation of thermoregulatory responses to changes in environmental temperature, although central effects have also been described (10, 18, 20–23). Interestingly, differences in Tc levels between sexes are mostly eliminated in gonadectomized adult mice (10), suggesting that sex effects on Tc are mainly mediated by gonadal sex hormones.

**Figure 3:**
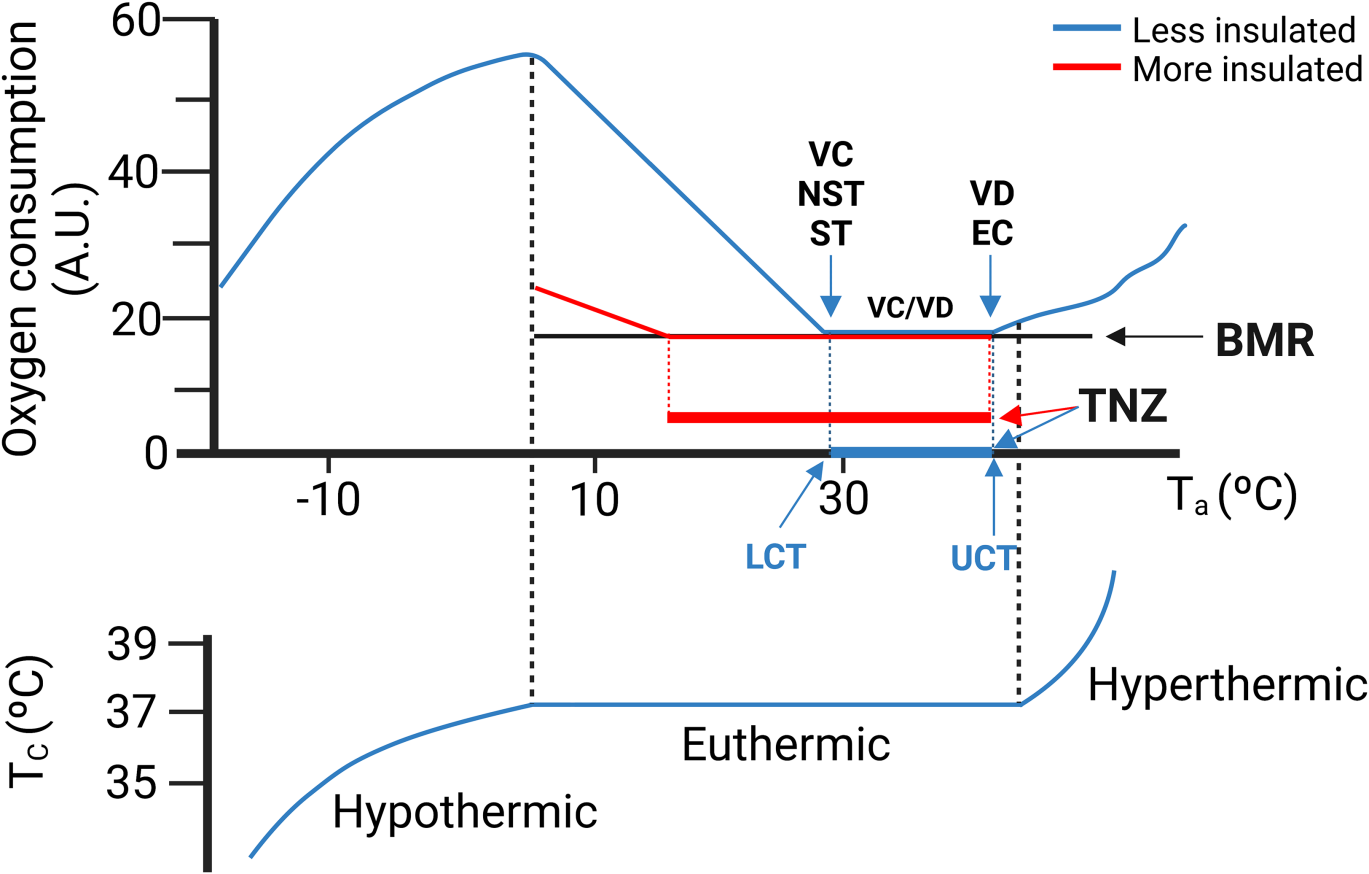
(Upper graph) A theoretical ambient temperature-metabolism plot. Thermoneutral zone (TNZ) is defined as the range of ambient temperature (Ta) at which core temperature (Tc) regulation is achieved only by obligatory thermogenesis and sensible heat loss. The lower critical temperature (LCT) represents the lower limit of the TNZ. Below the LCT, facultative heat production is activated to maintain thermal balance. The upper critical temperature (UCT) represents the upper limit of the TNZ. Above the UCT thermal balance is maintained by vasodilation and evaporation. Note that a more insulated animal (in red) presents lower LCT and wider TNZ. (Lower graph) Core temperature (Tc). (Lower graph) Once the thermoregulatory mechanisms fail, the organism will not be able to maintain its Tc. In humans Tc is rather stable. In contrast in mice Tc is much more variable and TNZ is almost a discrete temperature point (24, 25). NST, non-shivering thermogenesis; ST, shivering thermogenesis; VD, Vasodilation; EC, Evaporative cooling. BMR, Basal metabolic rate. Modified with permission from Cannon et al., 2011 (26).

In all mammals there is a range of environmental temperatures within which basal metabolism generates enough heat to maintain Tc. This temperature interval is known as the thermoneutral zone (TNZ) (**Figure 3**). When environmental temperatures either exceed or fall below the TNZ, the metabolic rate increases by activation of thermoregulatory mechanisms. Above the TNZ, (above the upper critical temperature, UCT), there is an increase of the metabolic rate because of the energy cost of the mechanisms for cooling. Conversely, at the lower bound of the TNZ (lower critical temperature, LCT), the animal activates facultative heat production mechanisms to defend its body temperature. For most research animals (and for naked humans), LCT lies close to 30 °C. The increase in heat production required to balance heat lost is inversely proportional to environmental temperature (**Figure 3**). The slope of the line is a measure of the thermal conductance, i.e., steep for poorly insulated animals (e.g., mice, naked human) and a shallow slope for good insulation (e.g., arctic species, dressed humans). The TNZ will therefore depend principally on basal thermogenesis, body size and insulation. Greater basal thermogenesis, greater insulation and greater sizes produce larger TNZs. In contrast, smaller animals present a greater surface area-to-volume ratio and are more susceptible to heat loss.

Sex also influences the TNZ in rodents and humans. Experiments with rodents have demonstrated a higher threshold temperature for cold-induced thermogenic response in females compared to males (27). Likewise, in humans, activation of thermoregulatory mechanisms in response to cold and heat are shifted to higher temperatures in women in relation to men (28–31). Thus, it was shown that in lightly dressed women, metabolic heat production increases when air temperature decreases below 31 °C, while in men the LCT was found at 28.5 °C (32). More research is warranted to determine whether the width of the TNZ is also different between sexes. Notice, that in many human studies, data refers to the range of internal body temperature between thermoregulatory thresholds (Core interthreshold zone, CIZ), and not to the ambient temperature range, as it defines the concept of TNZ.

Sex-dependent anthropometric parameters contribute to the observed differences in TNZ between sexes. In general, female mammals are smaller than males and have a larger surface-to-mass ratio, which favours heat loss. In contrast, females have more fat content than males (both whole body and subcutaneous), which increases insulation. Differences in obligatory thermogenesis have also been described. Women present lower levels of basal metabolic rate (BMR) than men (a reduction of about a 23%) (13, 33), even at a given body mass, although the difference is much smaller (about 3%) (33). But the most important factor for the higher threshold temperatures of thermoeffector responses in females is progesterone. Accordingly, the elevated levels of this sexual hormone in the luteal phase in women (**Figure 1**) are responsible for the shift of most thermoregulatory response thresholds towards warmer temperatures during this phase in relation to the follicular phase, as we will detail below (6, 18, 31, 34, 35).

Because we often use mice to model human physiology, we need to be aware that mice and human exhibit large differences in body size, shape and composition, which impact on thermal homeostasis. In mice, thermal conductance is higher, Tc is more variable and unstable, temperature preference is set at warmer temperatures and metabolic rate is greatly enhanced when compared to humans (24). In typical housing conditions of 21-24 °C, mice are subjected to a situation of chronic cold stress, even more intense in the case of females. This will affect the experimental outcomes and the possible interpretation of the results. Considering that humans live most of their time close to or in thermoneutral conditions (due to clothing and to the control of housing temperature), numerous authors have recently claimed that mice should be housed at thermoneutrality, to develop a more humanized experimental model. Nevertheless, while humans can maintain a constant Tc across the range of temperatures which are considered as the TNZ (between the LCT and UCT) (**Figure 3**), Tc rises in mice, especially over 30 °C (24, 25), with a circadian variation. This near absence of thermoneutrality in mice together with the higher values of LCT in females than in males make very complex to establish standard conditions of ambient temperatures for housing mice. Setting an external temperature of 26-28 °C, incorporating abundant bedding and nesting material in mouse cages (especially for females) and controlling housing density will help mice to have a more active role in their thermoregulation under a thermal situation more similar to humans.

## 3 Sex differences in thermosensation and thermoregulation

### 3.1 Thermosensation and thermal preference

Mammals sense innocuous and noxious ambient temperatures through the firing of different subpopulations of cutaneous thermosensitive neurons. This information is relayed to the brain *via* ascending projections to sensory cortical areas and to the hypothalamus to serve a dual purpose, evoke conscious perception of temperature changes and thermoregulate (4, 36).

Cold and warm thermoreceptors convey information about non-painful temperatures and show rapid adaptation. In contrast, cold and heat nociceptors convey noxious temperatures and mostly do not adapt (37). However, even at the peripheral level, the processing of thermal signals is complex (38). Skin non-neuronal cells (e.g., keratinocytes) also communicate with sensory endings and play a role in sensing thermal stimuli (39).

Transient receptor potential (TRP) cation channels have been identified as the key molecular sensors responsible for cutaneous thermosensation (40, 41). Their activation, in coordination with other ion channels, convert thermal stimuli into propagated electrical activity. There are several channels in the TRP family gated by temperature. Collectively their activity covers a broad range of biologically relevant temperatures. TRPV1, TRPV4, TRPM2, TRPM3, TRPM8, TRPC5, and TRPA1 are expressed in primary sensory neurons. In addition, TRPC5, TRPV3 and TRPV4 are expressed in non-neuronal skin cells. However, the contribution of these last two TRP channels to thermosensation is not clear; TRPV3/TRPV4 double KO mice do not have behavioural deficits to thermal stimuli (42).

TRPV1-expressing primary sensory neurons play an essential role in acute noxious heat sensing and heat-induced pain (43). Nevertheless, TRPV1-deficient mice still maintain clear noxious heat responses (44–46), suggesting the existence of additional heat sensors. Thus, TRPV1, TRPM3 and TRPA1 show an overlapping pattern of expression and their combined ablation is required to abolish noxious heat sensation in mice, indicating some functional redundancy (47). The specific contribution of each channel to acute noxious heat sensing is probably dynamic and influenced by the metabolic status of the tissue.

Considering cold thermosensation, TRPM8 has been identified as the principal sensor of environmental cold (48). Behavioural experiments indicate that TRPM8 is essential for the detection of mild (i.e. innocuous) cold temperatures (48) and it also plays a role in noxious cold sensing, although in TRPM8-deficient mice noxious cold detection is impaired, but not abolished (49). While the majority of TRPM8 expression is found in peripheral sensory neurons, there are also TRPM8-expressing neurons in the rodent brain, especially in areas related to autonomic thermoregulation, including the preoptic area of the hypothalamus (POA) and in areas of the limbic system (50). On the other hand, almost twenty years ago, TRPA1 was identified as a noxious cold sensor in primary sensory neurons, activated by temperatures below 17 °C (51). However, the role of TRPA1 in thermosensation is still poorly defined (37). Different results indicate activation of TRPA1 by heat and cold, depending on cellular context and metabolic factors including the presence of reactive oxygen products.

Central integration of thermoafferent information establishes not only a thermal sensation, but also a perception of pleasure or displeasure. Thus, thermal comfort refers to the satisfaction with the thermal environment. Thermal comfort or discomfort is important for defending core body temperature because it is considered as the principal driver for thermal behaviour. The ambient temperature range associated with thermal comfort (Thermal comfort zone, TCZ) is narrower than the TNZ (52). Considering that animals seek for thermal comfort, behavioral thermal strategies are probably initiated even before ambient temperature reaches the limits of the TNZ.

Many studies support that sex, body shape and composition, and age are the most important factors influencing thermal perception and thermal preference (53–55). Sex differences in sensitivity to cold and warm temperatures exist in rodents as well as in humans. Females display lower thresholds for cold and warm sensation (i.e. detect smaller changes) (56, 57), and report a stronger sensation to a fixed warm stimulus (58). In addition, women prefer higher ambient temperatures and experience greater thermal discomfort, especially in cooler conditions (59). Thus, cool temperatures (18 °C to 22 °C) are found uncomfortable for women, but acceptable for men. Conversely, women find comfortable higher temperatures that for men are intolerably warm. The mechanisms responsible for sexual differences in thermosensation and comfort are still unclear. This variability may arise from differences in the physiology of primary sensory neurons of males and females and/or in the integration of the afferent signals at the central level. Thus, two studies have reported that testosterone constitutes a fine regulator of thermosensitivity, through its interaction with TRPM8 ion channels (60–62). The higher levels of testosterone in males would induce desensitization of TRPM8 channels, making males less sensitive to mild cold. Also, it has been described a marked sexual dimorphism in cold sensitivity in transgenic mice overexpressing the glucocorticoid-regulated kinase 1 (SGK1.1), a regulator of the M potassium current. Hyposensitivity to noxious cold stimuli was only observed in male mice (63). The specific mechanism underlying this differential phenotype was not explored.

The preference of females for warmer ambient temperatures might also be due to central mechanisms controlling whole body thermal homeostasis, i.e., sex-dependent control of the temperature set point(s) in the brain. Interestingly, gonadectomy in adult life does not change thermal preference of male and female mice (64). Therefore, other sex-specific factors, rather than gonadal hormones are responsible for the sex-dependent regulation of this set point(s) in the POA, or alternatively, this central set point is established before puberty. Nevertheless, there is some evidence suggesting that thermal preference in women could change across the ovarian cycle (6, 65), which would indicate a hormonal control. More investigation is required to evaluate the sexual regulation of the thermal comfort zone.

### 3.2 Generalities of the main thermoregulatory mechanisms

When ambient temperatures exit the TNZ, the thermoregulatory centre in the POA sends appropriate output signals to peripheral effectors. Most studies have been centered on autonomic mechanisms, but complex behaviours with thermoregulatory consequences also play an important role in body temperature regulation.

In general, mammals adopt specific behaviours to keep themselves into the TNZ. They are collectively called thermal behaviours or behavioural thermoregulation and are initiated to minimise energy expenditure while defending Tc (66). Common thermal behaviours include thermotaxis (movement towards or away from heat), postural changes (increasing or reducing exposed surface area) and creating new environments (huddling or nesting) (66–68). In humans, dressing, living in houses and switching heating or cooling systems play a major role. Mammals also increase or decrease food consumption and locomotor activity to maintain thermal balance in cold and warm environments, respectively (3, 69, 70).

In addition to behavioural changes, autonomic responses are launched to alter heat exchange with the environment. Heat loss occurs through different mechanisms, i.e., dry or sensible heat loss (conduction, convection, radiation) and evaporation. Peripheral vasoconstriction and reduced blood flow to extremities, such as the tail and paws in rodents and hands and feet in human, is an efficient mechanism to reduce heat loss in cold environments (66). In contrast, peripheral vasodilation, or increased blood flow to extremities releases body heat in response to a warm ambient. As the external temperature exceeds the UCT, evaporative heat loss is also activated and greatly contributes to heat dissipation. Differently to the dry heat loss, evaporative heat dissipation constitutes an energy costly process, due to the energy required for water vaporization. Respiratory evaporation by panting (71) or saliva spreading (72) increases evaporative heat dissipation in many mammals, while the development of evaporation by sweating plays a central role in human thermoregulation.

In cold environments, behavioural and autonomic heat-retaining responses are frequently not sufficient to maintain Tc, and facultative thermogenic mechanisms are recruited. Thus, skeletal muscle shivering is rapidly activated as a thermogenic response to cold. If low temperature is persistent, shivering thermogenesis will be substituted by non-shivering thermogenesis of brown adipose tissue (BAT), an adaptive mechanism which develops over weeks (26).

### 3.3 Heat-conserving and heat-dissipating mechanisms

The cardiovascular system in mammals constantly pumps warm blood, transferring heat from internal organs to the skin, where changes in cutaneous circulation can regulate whether heat is dissipated or conserved, to maintain a stable Tc. Cutaneous vasomotor control constitutes a first-line thermoregulatory defense, also in the TNZ.

The cutaneous blood flow is regulated differently in the non-glabrous (hairy) and glabrous (non-hairy) skin. In the hairy skin of humans, the sympathetic nervous system is composed of two different branches: the sympathetic noradrenergic vasoconstrictor system, which displays tonic activity in normothermic conditions and a cholinergic active vasodilator system, that is stimulated only upon warming. In contrast, the non-hairy skin (e.g., hands and feet of humans, tail of rodents) is heavily vascularized, displaying numerous arteriovenous anastomoses and with a dense innervation of the sympathetic noradrenergic vasoconstrictor system, but without an active vasodilator system; blood flow regulation depends entirely on the sympathetic vasoconstriction. The large surface-to-volume ratio of these areas permit rapid and efficient heat loss or heat conservation as needed (73, 74).

#### 3.3.1 Heat conserving response

Cooling of the skin activates cutaneous cold thermoreceptors that initiate the vasoconstrictor response. Local and reflex central mechanisms interact in this process. In both cases, the adrenergic sympathetic system plays a central role (75). Furthermore, when the cold stimulus is prolonged, the cutaneous vasoconstriction abruptly stops and the vessels dilate, allowing the reperfusion of the tissue to avoid cold-induced tissue injury; a process called cold-induced vasodilation (CIVD). Some studies have involved TRPM8 in skin vasomotor control in rodent tail or paws in the context of thermoregulation (70, 76, 77). In addition, there is evidence that TRPA1 channels are required for cold-induced vasoconstriction and CIVD in paw cutaneous vasculature (77, 78), although the involvement of TRPA1 in cold sensation and thermoregulation is contentious.

#### 3.3.2 Heat dissipating mechanisms

In response to an increase in Tc or the exposure to a hot environment, the thermoregulatory system activates two main effector mechanisms to dissipate heat to the environment and maintain thermal homeostasis. The first response is the activation of cutaneous vasodilation, and if this process is insufficient to maintain core body temperature stable, sweating, a more energetically expensive mechanism, is activated.

Cutaneous vasodilation is promoted *via* two mechanisms: the inhibition of the vasoconstrictor component of the SNS and, the activation of the vasodilator component of the SNS (also called cutaneous active vasodilation). Two members of the thermosensitive Transient Receptor Potential Vanilloid subfamily, TRPV1 and TRPV3 seem to mediate heat-induced cutaneous vasodilation in mice and humans (79–81). The activation of these channels stimulates the release of vasodilators such as acetylcholine and CGRP (78–81).

When cutaneous vasodilation is unable to meet the thermal homeostatic requirements set by the POA, the SNS stimulates sweating by releasing acetylcholine which binds to muscarinic type-3 (M_3_) receptors expressed in the sweat glands (82). The evaporative cooling of sweat lowers the skin temperature. Of the three types of sweat glands (eccrine, apocrine, and apoeccrine), only eccrine glands are involved in thermoregulation and are only present in apes and humans (82). Humans have a much higher gland density than apes (about 10 times higher) and furry mammals, including our closest primate relatives, rely mainly on panting to increase their evaporative heat loss. In contrast, sweating constitutes the most important thermoregulatory mechanism in human species above the UCT (upper critical temperature) and apparently could have played an important role in driving human evolution (83).

Some studies have evaluated the effect of sex dimorphism and sexual hormones on the vasomotor and sweating response to a thermal challenge, but many gaps in knowledge still exist. The use of different experimental settings has yielded confusing and even contradictory results. Regarding to heat conserving mechanisms, some authors have described that women display a greater activity of the SNS innervating cutaneous vessels and a greater skin vasoconstriction in response to cold, especially in the luteal phase (84–86), while others found a lower SNS activity and a lower (87) or similar (88) cold-induced vasoconstriction in comparison to men. Furthermore, a similar cold-induced vasodilation (CIVD) was observed in both sexes (85, 89), or, conversely, a higher frequency of CIVD events was detected in women (90).

Considering responses to heat or exercise, most evidence suggest similar vasodilatory responses between both sexes (91, 92), although differences have been reported in NO and COX-PGE signalling in cutaneous blood flow (93–95). In relation to sweating, some sex dimorphisms have been described. On average, women have more sweat glands and higher sweat gland density than men (96). However, during passive and exercise heat stress, women exhibit a lower sweating rate than men (97, 98). Evidence suggest that sweat glands in women have a lower frequency and amount of sweat excretion than men (97), which may be due to lower acetylcholine-induced sweating responses (99). In all, modest differences in the ability to thermoregulate and acclimate to heat challenges were observed between the sexes, only evident during exercises of very high intensity and in environmental contexts with very heavy demands for heat loss (91).

**Figure 4:**
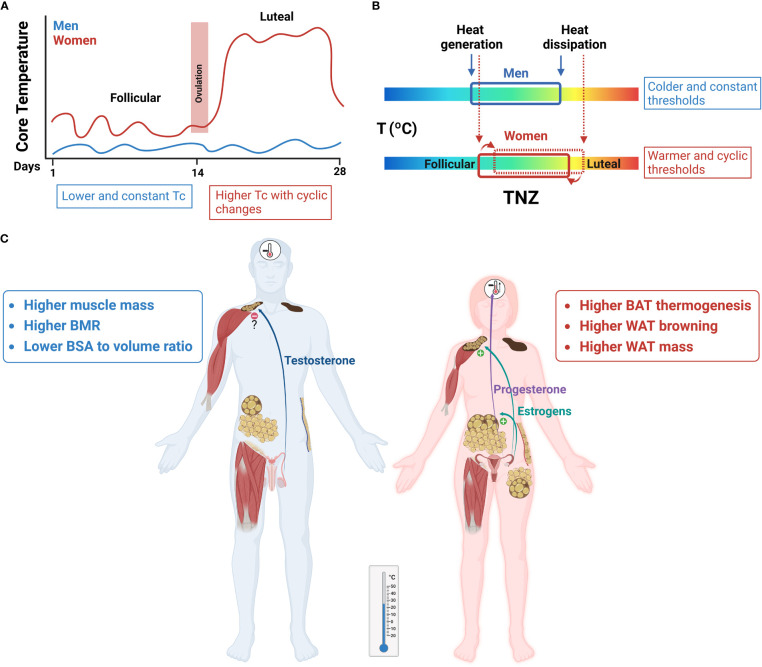
Sex differences in thermoregulation have an impact on energy homeostasis. Red and blue represent women and men, respectively. **(A)** Core temperature (Tc) levels are higher in women than in men and vary across the ovarian cycle, associated to changes in progesterone:estrogens circulating concentrations. In the luteal phase there is an increase of ≈ 0.5 °C, when compared to the follicular phase. **(B)** A representation of the thermoneutral zone (TNZ) in both sexes. The increase of Tc levels in women is a regulated process, as shown by the shifts in thermoregulatory thresholds of effector mechanisms toward the defense of this higher temperature. Accordingly, cold output mechanisms will be activated at higher ambient temperatures in women than in men. **(C)** At mild temperatures a greater BAT and WAT browning thermogenesis will be observed in women compared to men, which will increase energy expenditure. Sexual hormones play a central role in these sex differences, with estrogens as principal stimulators of BAT activity and WAT browning and regulators of vasomotor control, and progesterone as a thermogenic signal acting at the set-point(s) in the hypothalamus.

Sexual hormones and the ovarian cycle regulate heat conserving and heat dissipating processes, although the mechanisms are still not well understood and merit further investigation. Most studies show that estrogens promote vasodilation by direct effects on skin blood vessels, but also acting in the CNS (18). In the case of progesterone, its major function seems to be the change of the thermoregulatory set points of all heat conserving and heat dissipating mechanisms towards warmer temperatures (i.e., cutaneous vasoconstriction during cooling, and cutaneous vasodilation and sweating during warming). This shift has been clearly shown in the luteal phase of women in relation to the follicular phase (6, 23, 97, 100, 101) (**Figure 4**).

More research is needed to understand the thermoregulation adjustments in women after menopause, and the mechanisms responsible for the vasomotor symptoms (hot flashes) (18).

### 3.4 Thermogenic mechanisms

Shivering and non-shivering thermogenesis are the two major facultative thermogenic mechanisms in mammals. Shivering thermogenesis (ST) generates heat by contractions in the skeletal muscle (102). This type of thermogenesis is very effective in large mammals like humans, but it is less efficient in rodents due to their relatively small muscle mass (66). Instead, rodents rely more on non-shivering thermogenesis (NST), which takes place in the brown adipose tissue (BAT) and is also referred as BAT thermogenesis. NST in humans is not exclusively produced in BAT. Muscle-derived NST is an emerging topic, although still poorly investigated (103).

#### 3.4.1 Shivering

Shivering is a very potent and rapid process to produce heat in response to a cold environment, through the involuntary contraction of skeletal muscle. The production of heat derives from the enthalpy of ATP hydrolysis that is not used by the machinery of the muscle cell to perform mechanical work (104–106). Although the neural circuitry responsible for the shivering response is not fully understood, it has been proposed that it depends on the activation of somatic premotor neurons located in the rostral medullary raphe (4), resulting in the excitation of spinal somatic motor outputs driving shivering. Interestingly, these premotor neurons can also activate sympathetic outputs in the spinal cord, thus providing a common neural pathway for shivering and non-shivering thermogenesis (107).

Few studies have evaluated sex differences in shivering in experimental animals. Most of the information comes from studies in humans and a major determinant of the ambient temperature at which shivering is activated is sex. In women shivering begins at higher temperatures than in men, even when comparing persons of both sexes with similar anthropometric characteristics (54). Also, although women seem to display a similar shivering rate than men, they oxidized more lipids at the same shivering intensity (108). Most studies also report the influence of the ovarian cycle in the initiation of cold-induced shivering, with the set-point moving towards warmer temperatures in the luteal phase (6) (**Figure 4**). Nevertheless, no changes in shivering activity, substrate utilization or oxidation rates were observed between both ovarian phases (108).

#### 3.4.2 BAT thermogenesis

The process of heat generation in BAT is fuelled by a mitochondrial protein, uncoupling protein 1 (UCP1), that is expressed in brown or beige adipocytes, and its function is to uncouple the respiratory chain from ATP synthesis, transforming the lipids accumulated in BAT into heat (109, 110). Activity and expression of UCP1 and therefore BAT thermogenesis is regulated principally by the noradrenergic system. On one hand, noradrenaline (NA) favours brown adipocytes differentiation, cellular proliferation and mitochondriogenesis, increasing UCP1 levels (110–114). On the other hand, it also promotes lipolysis, a controlling step in BAT thermogenesis, because released FFAs are positive UCP1 modulators (115–117). In brown adipocytes different types of adrenergic receptors are expressed, i.e., β-adrenoreceptors (β1-AR, β2-AR, and β3-AR), which activate adenylyl cyclase; α2-AR, which inhibits adenylyl cyclase (115, 118). The existence of all these types of AR in brown adipocytes reflects the complexity of the regulation of BAT activity. In addition to the relative affinity of each receptor subtype for NA (α2>β1≥β2>β3), their absolute levels are also functionally relevant. In rodents, β3-AR is the most highly expressed adrenoreceptor in brown adipocytes (116). This subtype is also the most important in the stimulation of UCP1 synthesis and in NA-induced lipolysis (119–122). However, it has been shown that the balance between α2 and β3-AR is crucial in the regulation of these processes (123).

In rodents, sex dimorphism has been extensively described in brown adipose tissue thermogenic capacity and in the response to cold (27, 124). Thus, although at an ambient temperature of 28 °C, levels of UCP1 are similar between male and female rats, at colder temperatures (22 °C and 18 °C) UCP1 levels are significantly higher in females. Therefore, at the usual rodent housing temperature (22 °C), BAT in female rats represents a greater proportion of total body mass, shows more hypertrophy and a more multilocular pattern of lipid droplets and exhibits higher UCP1 levels and larger and more functional BAT mitochondria. Of note, at 4 °C both sexes display similar maximal values of UCP1 (27), suggesting sex-specific thresholds of BAT stimulation, with females activating this thermogenic mechanism at warmer ambient temperatures than males. Adrenoreceptor protein levels also vary between sexes. Female rats display a lower α2A/β3-AR ratio than male rats, especially due to a reduced content of α2A-AR (120, 124), which supports the increased mitochondrial function and metabolism in female brown adipocytes.

BAT has received increasing attention following the finding of active BAT in adult humans. Previously, it was thought that BAT was only found in hibernating animals, small mammals, and human infants. However, studies using principally positron emission tomography/computed tomography (PET/CT) disclosed the presence of active BAT in healthy adults, principally in the cervico-supraclavicular area, but also in axillary, mediastinal, paravertebral, perirenal, and peri-aortic regions (125–130). A higher BAT prevalence and activity has been described in women than in men (128, 131–136), although some reports show conflicting results (137). Furthermore, sexual differences have also been observed in the distribution of BAT depots (138), adding more complexity to the investigation of BAT physiology in humans. With increasing age, BAT activity declines (133, 139, 140), but the reduction appears to be greater in men (133).

Sex hormones play a critical role in the development of sex differences in BAT thermogenic activity (141–143). Estrogens induce a stimulatory effect on BAT activity and are among the most significant regulators of BAT function and differentiation (143). In addition to the direct effects on brown adipocytes, they also enhance BAT activity and thermogenesis *via* the brain, especially the hypothalamus, leading to an activated sympathetic nervous system (144). Thus, brown adipocyte proliferation and differentiation, BAT thermogenic activity and UCP1 expression, and levels of the adipokine BMP8B are increased by estrogens (145–150). The effects of androgens on BAT activity are more difficult to unravel, although most results show that androgens appear to have an inhibitory effect (147, 148, 151–157).

In humans, the studies of sex hormones in BAT physiology suggest comparable effects to those reported in rodents, although more investigation is required. Thus, sex dimorphism in BAT activity is reduced when women become postmenopausal, suggesting that the age-related decline in circulating levels of sex hormones may contribute to the loss of BAT activity (158–160). One important aspect that still needs to be sorted out is the importance of the canonical β3-signalling in human BAT *per se* and the existence of sex differences in the adrenergic innervation of this organ. Finally, although the study of BAT activity during the ovarian cycle should be addressed with more detail, evidence has shown that basal BAT temperature increases during the luteal phase and this increase is positively correlated with progesterone levels. Furthermore, the adaptive increase of BAT temperature in response to cold is greater in women than in men, irrespective of the ovarian phase, and positively correlated with estradiol levels (161).

In addition to the cold-induced activity of BAT, brown adipocytes are also activated in mammals by overeating with high fat and/or high carbohydrate diets. This process, called diet-induced thermogenesis (DIT), involves a stimulation of the sympathetic innervation of BAT and favours burning excess calories, with a potential value for obesity and its comorbidities (26, 162). Interestingly, the increase in BAT temperature in response to a meal is also greater in women than in men (161).

Finally, cold temperature, but also other stimuli (e.g., exercise, several hormones, and cytokines) can induce a metabolic remodelling of WAT, with the appearance of multilocular beige adipocytes, an increased expression of thermogenic and mitochondria-related genes, particularly UCP1. The process is called as browning of WAT and has also received great attention for the treatment of obesity and metabolic disorders, as these “beige” fat depots might contribute to increase energy expenditure and energy partitioning (163, 164). Although browning of white fat is clearly found in rodents, “beiging” susceptibility has been questioned in human WAT. Nevertheless, accumulating evidence suggests that browning of WAT cells also occur in humans, i.e., conditions with potent adrenergic innervation like cancer-associated cachexia (165), or some pharmacological compounds show an intense browning of WAT (164). More investigations are required to fully characterize human WAT depots and identify human fat cell populations that give rise to beige fat cells. Interestingly, both rodent and human studies have shown a higher capacity for WAT browning in females (166–168).

## 4 Impact of thermoregulation on energy homeostasis: Importance of sex

Body weight and body composition result from the equilibrium between food intake and energy expenditure. Energy homeostasis, energy metabolism and energy deposition appear, at first sight, very simple and direct processes, but they constitute entangled conditions. Numerous factors (i.e., genetic determinants, sexual hormones, environment, nutritional characteristics, hedonic behaviour, grade of activity, metabolic efficiency, social and economic factors, etc.) influence and regulate the different components of this balance. Although research has focused primarily on either increased palatable food intake and the effects of sedentary lifestyles, this paradigm may not fully address the complexity of interactions that modulate both the food that individuals eat, nor the processes that account for energy expenditure and energy storage. In this regard, it has been suggested that multiple, undervalued, or subtle factors may be additionally contributing to the pandemic increase in fat gain. As discussed below, one of these may be ambient temperature and the thermal mechanisms involved in maintaining homeothermy (169–171). Furthermore, sexual differences in thermal physiology influence energy homeostasis.

Ambient temperature and thermoregulation have a major role on energy balance by regulating food intake, heat production and energy expenditure. In fact, mammals dedicate 40-50% of their total energy expenditure to sustain a constant Tc at 37 °C (171). Among the thermoregulatory processes, BAT thermogenesis plays a central role in the regulation of both, body temperature and energy homeostasis. Thus, a diminished response of BAT to food intake and to cold temperature has been observed in practically all genetic forms of obesity (172). In recent years, BAT has received renewed interest due to its lipid oxidizing capacity. BAT and browning of WAT have become attractive therapeutic targets to increase energy expenditure and clearing excessive fat from circulation. Thus, activating thermogenic metabolism in BAT and WAT appears to exert a beneficial impact in obesity, type-2 diabetes, dyslipidaemia and cardiovascular pathologies, improving the glucose and lipid homeostasis (136, 173–177).

Interestingly, most mammals spend most of their time in ambient temperatures lower than their TNZ, which implies that they activate energy-costly thermoregulatory mechanisms. In contrast, in the last decades, humans have greatly increased the time spent indoors, with a widespread access to central heating and air conditioning and higher expectations of thermal comfort. All these factors contribute to reduce mild thermal stress. More time spent within the TNZ has been associated with increased obesity rates (169, 170), presumably through reductions of energy expenditure due to a decrease in thermogenesis, including a diminished efficiency in diet-induced thermogenesis.

When analyzing the effect of ambient temperature on energy homeostasis, the other variable to consider is energy intake. Food intake is required to meet physiological needs of nutrients, but also as a source of energy, including what is needed for thermal homeostasis. An inverse relationship exists between ambient temperature and food intake across a wide range of temperatures in multiple species, including humans. During short exposures to cold, an almost perfect fit is shown, with intake adjustment compensating accurately for temperature-induced changes in expenditure. In contrast, during long-term cold exposures, the rise in food intake is insufficient to compensate for the increased metabolic output (169, 178, 179), resulting in a progressive reduction of fat mass. Conversely, when environmental temperatures get warmer and close to the TNZ, intake is progressively suppressed, but this reduction is lower than the decrease observed in energy expenditure, particularly in the case of highly tasty foods (169). This has important implications in our ‘obesogenic’ society, living most/all our time in our TNZ range of temperatures and surrounded by food, which is always accessible and highly palatable. The contribution of the environmental comfort of life to the current obesity pandemic merits further investigation. Furthermore, the study of the metabolic effects of cold ambient temperatures, including the activation of cold thermosensors is an emerging field with great physiological and medical interest. Thus, accumulating evidence indicate that mice exposition to cold temperatures induce loss of fat mass in animals fed a standard or a high fat diet, with a significant activation of BAT and WAT browning (178, 179). Contrarily, mice deficient in TRPM8 (the main cold sensor) develop obesity when housed at mild temperatures, exhibiting diurnal hyperphagia, reduced lipid utilization (70) and an altered circadian physiology (14). Furthermore, oral or topic TRPM8 agonists have proven to be effective in reducing WAT in mice (180–183). Therefore, targeting temperature-induced signalling pathways may open new therapeutic avenues in obesity and its metabolic comorbidities. Furthermore, sustained alterations in the function of certain thermoTRP channels (e.g.,TRPM8 polymorphisms) may play an unrecognized role in obesity pathogenesis.

Sex differences in thermoregulation have an impact on energy homeostasis, that needs to be considered. As discussed above, the neutral interval of temperatures is shifted to higher temperatures in female mammals (including women). Furthermore, in the feminine sex the TNZ experiences cyclic changes according to the progesterone:estrogens plasma levels, with higher temperature set-point(s) in the luteal phase. A shift of the TNZ towards higher temperatures implies that cold-induced mechanisms will be activated at warmer temperatures, as it has been widely demonstrated in rodents and women. Consequently, the metabolic impact of cold temperature on energy balance will be observed at higher ambient temperatures in females than in males. Indeed, a recent study showed that housing female mice for two weeks below 25 °C induced a decrease in fat mass, while only temperatures below 18 °C produced a reduction of white adipose tissue in males (179). Further research is warranted to investigate in humans the effectiveness of chronic stimulation of cold temperature signalling pathways on fat loss, glucose and lipid homeostasis and evaluate the threshold differences between both sexes.

Although obesity is increasing in both sexes, obesity rates and associated comorbidities are different between women and men. Data from the Centers for Disease control and prevention (CDC, USA) and from the World Health Organization (WHO) reveal that women have less prevalence of overweight than men, lower/similar rates of obesity, but double the risk to develop extreme obesity, especially after menopause (https://www.cdc.gov/obesity, https://www.who.int/health-topics/obesity). Genetic factors and sexual hormones determine significant differences in fat mass and distribution between sexes. Thus, women have a greater proportion of body fat mass than men, intimately linked to reproductive function, with larger depots of subcutaneous fat (with thermoregulatory value). In contrast, men tend to gain more visceral fat, which is associated with an increased risk of cardiometabolic complications (184). Interestingly, it has been described that adipose tissue has higher metabolic rate per kilogram in women, which agrees with the initiation of cold-induced thermoregulatory mechanisms at warmer temperatures and the increased expression of genes involved in mitochondrial function (168). Although the contribution of fat to the basal metabolic rate (BMR) is low, because the metabolic rate of adipose tissue is much lower than other internal organs and the skeletal muscle, a small increase in fat metabolism may have important long-term implications for conditions and diseases that develop slowly over several decades, like obesity and its health consequences (**Figure 4**). Lifestyle changes affecting thermoregulation including increasing time spent in outdoor activities, a greater exposure to seasonal cold, choosing walking or cycling instead of temperature-controlled car transportation may cooperate with a healthy food intake and exercise in preventing and reducing obesity, especially in women. In addition, the potential use of cold-mimetic compounds may override the problems associated to the discomfort of cold exposition protocols. More investigation is needed to develop new TRPM8 agonists, and study in humans their long-term effects on body fat and metabolism, paying attention to differences between sexes. Finally, although indoor temperatures are often tightly set at high fixed values, some recent studies have approached the potential application of moderate temperature drifts or ramps towards colder values to increase energy expenditure, while maintaining thermal comfort. The combination of adding more temperature variability in buildings with the use of personal comfort systems by selecting heating or cooling individual body parts to improve whole body thermal comfort is under active investigation (185, 186). Differences in the thresholds of TNZ and thermal comfort between sexes need to be considered in this type of studies (**Figure 4**).

## 5 Conclusion

Increasing evidence shows that differences in thermoregulation between sexes may play an important role in the observed sex differences in energy regulation, body weight and body composition. Although our knowledge of the interrelationship between thermal and energy homeostasis has greatly increased, most experimental studies in mice and humans examine only individuals of one sex, mainly males, to reduce the total number of subjects and the cost. Males are preferred for simplicity. There are, however, obvious downsides by omitting the feminine sex, from lack of knowledge of the physiology of females (50% of total population) to implications for optimal therapeutic impact and implementation of the findings depending on the sex. Understanding how ambient temperature and cold sensors, like TRPM8, impact on our metabolism is very relevant from a scientific, pharmaceutical, and medical point of view. It opens the opportunity for novel public health initiatives to address obesity and new therapeutic strategies. To establish in a rigorous way the influence of thermoregulation on energy homeostasis and the biomedical basis for better personalized treatment options, it will become crucial to carry out detailed and more standardized studies on molecular and functional sex differences, considering the ovarian cycle and the menopause.

## Author contributions

RS: designed review; CF-P and AR: performed investigation; RS, CF-P, AR, FV, and VA: writing, reviewing and editing; CF-P, AR, VA, and RS: Visualization; RS and FV: Funding acquisition. All authors contributed to the article and approved the submitted version.
